# Vitamin D in male reproduction: molecular mechanisms and pharmacological perspectives

**DOI:** 10.3389/fphar.2026.1849299

**Published:** 2026-09-03

**Authors:** Jing Liu, Bang Liu, Xingyun Qiu, Mengmei Lv, Shasha Liu, Dai Zhou

**Affiliations:** 1 Hunan Provincial Key Laboratory of Regional Hereditary Birth Defect Prevention and Control, Changsha Hospital for Maternal & Child Health Care Affiliated to Hunan Normal University, Changsha, Hunan, China; 2 The Third School of Clinical Medicine, Ningxia Medical University, Yinchuan, Ningxia, China; 3 Hainan Provincial Key Laboratory for Human Reproductive Medicine and Genetic Research, Hainan Provincial Clinical Research Center for Thalassemia, Key Laboratory of Reproductive Health Diseases Research and Translation (Hainan Medical University), Ministry of Education, The First Affiliated Hospital of Hainan Medical University, Hainan Medical University, Haikou, Hainan, China

**Keywords:** male reproduction, molecular pharmacology, oxidative stress, sperm motility, vitamin D, vitamin D receptor (VDR)

## Abstract

Vitamin D has attracted increasing attention in male reproduction, male fertility, and sperm function, but its pharmacological relevance remains incompletely defined. This review synthesizes current evidence on the molecular mechanisms and clinical implications of vitamin D signaling in the male reproductive system. Available experimental and human data suggest that the vitamin D receptor (VDR) and vitamin D-metabolizing enzymes are present in testicular cells, the epididymis, accessory sex glands, and mature spermatozoa, supporting the possibility of both systemic and local actions. Mechanistically, vitamin D may regulate sperm capacitation and progressive motility through calcium-dependent non-genomic pathways. Ligand-activated VDR signaling may also contribute to antioxidant defense, mitochondrial stability, inflammatory control, steroidogenesis, germ-cell survival, and epigenetic regulation, although several of these pathways still require direct validation in human testicular tissue and spermatozoa. Clinically, low circulating 25-hydroxyvitamin D has been most consistently associated with impaired sperm motility, whereas associations with sperm concentration, total sperm count, reproductive hormones, and sperm DNA fragmentation index are less consistent. Interventional studies suggest that vitamin D supplementation may improve selected semen parameters in deficient men, but current trials do not support broad endocrine or fertility benefits in unselected populations. Importantly, emerging preclinical data indicate that excessive vitamin D exposure may impair spermatogenesis, highlighting a potential biphasic relationship in which both deficiency and excess are unfavorable. Thus, vitamin D should be viewed as a homeostatic modulator rather than a stand-alone treatment for male infertility. Future studies should define optimal therapeutic windows, clarify effects on sperm DNA integrity, and incorporate individual differences in VDR-related genetic and epigenetic regulation.

## 1 Introduction

### 1.1 The clinical challenge of male infertility

Infertility represents a significant global health burden, impacting an estimated 70–80 million couples and reflecting an overall prevalence of 8%–12% (Mascarenhas et al., 2012; Boivin et al., 2007). Male-specific issues are solely responsible for roughly 20%–30% of these diagnoses, while co-contributing alongside female factors in an additional 25%–40% of cases (Agarwal et al., 2015). The pathogenic origins of male infertility are highly diverse, driven by a combination of environmental, immunological, endocrine, and genetic determinants. Significantly, up to 40% of these clinical presentations are categorized as idiopathic, meaning that conventional diagnostic workups fail to reveal a definitive etiology (Punab et al., 2017; Agarwal et al., 2019). While routine semen quality assessments—evaluating sperm concentration, total count, motility, and morphology—are fundamental diagnostic tools, the correlation between these parameters and actual fertility outcomes is not strictly linear (Guzick et al., 2001). This persistent diagnostic and therapeutic gap underscores the urgent need to explore novel molecular mechanisms and potential pharmacological targets for male infertility.

### 1.2 Pharmacological profile and molecular metabolism of vitamin D

Vitamin D is a fat-soluble secosteroid primarily existing in two forms: vitamin D_2_ (ergocalciferol) and vitamin D_3_ (cholecalciferol) (Holick, 2007). In its parent form, vitamin D is biologically inactive and requires two sequential hydroxylation reactions to achieve its active pharmacological state (Christ et al., 2016). The first of these conversions is generally thought to occur in the liver, facilitated by 25-hydroxylase (CYP2R1) to form 25-hydroxyvitamin D_3_ [25(OH)D_3_]—often considered the primary serum indicator for clinical evaluations of vitamin D levels (Jones, 2008). Following this, a carefully regulated second hydroxylation usually happens within the renal system, where 1α-hydroxylase (CYP27B1) catalyzes the creation of the potent secosteroid hormone 1,25-dihydroxyvitamin D_3_ [1,25(OH)_2_D_3_] (Bikle, 2014).

Current consensus indicates that the physiological impacts of 1,25(OH)_2_D_3_ are largely mediated by its interaction with the vitamin D receptor (VDR) (Haussler et al., 2013). As a recognized member of the nuclear receptor superfamily, VDR is believed to pair with the retinoid X receptor (RXR) upon ligand attachment, forming a heterodimer that connects to specific vitamin D response elements (VDREs) to help orchestrate target gene transcription (Hanel et al., 2020). Although its classical roles are mainly associated with calcium and phosphorus regulation in the bones, kidneys, and intestines, the widespread detection of VDR across diverse human tissues indicates that vitamin D might engage in a much broader spectrum of non-classical, pleiotropic activities (Bouillon et al., 2008).

### 1.3 Bridging vitamin D and reproductive pharmacology

Over the past few years, the potential convergence of reproductive endocrinology and vitamin D pathways has drawn increasing attention from the pharmacological community (Cito et al., 2020). While its regulatory role in female reproductive health—such as in polycystic ovary syndrome, endometriosis, and assisted reproductive technology (ART) outcomes—is well-documented, its precise impact on male reproduction is an emerging Frontier (Miao et al., 2020). Initial investigations have noted the detectable presence of VDR across multiple male reproductive tissues, including the seminal vesicles, prostatic glands, epididymal tract, and testes (Chen and Zhi, 2020). Notably, researchers have also reported finding essential vitamin D-metabolizing enzymes alongside VDR within fully developed sperm cells (Arab et al., 2019). Such observations tentatively suggest the hypothesis that vitamin D might play a direct, localized role in orchestrating the molecular mechanisms of male reproductive operations (Cito et al., 2020). Despite these molecular clues, the specific pharmacological networks and the actual clinical benefits of administering vitamin D to infertile men are still viewed as highly debatable, often yielding inconsistent results among various observational cohorts and clinical trials (Arab et al., 2019).

### 1.4 Scope of the review

Moving beyond simple epidemiological correlations, this narrative synthesis intends to offer a cautious evaluation of the pharmacological prospects and underlying molecular pathways of vitamin D in the context of male fertility. We first aim to methodically review the localized presence of VDR and its corresponding metabolic machinery across various segments of the male reproductive tract (Cito et al., 2020). Furthermore, we will explore the precise molecular pathways through which vitamin D modulates sperm function, including calcium ion channel regulation, antioxidant defense mechanisms against oxidative stress, and steroidogenic gene regulation (De Angelis et al., 2017). Finally, we provide a critical appraisal of contemporary clinical trials, attempting to help reconcile the existing divide between fundamental molecular pharmacology and the practical utility of vitamin D therapies for infertile patients. In response to the growing translational interest in vitamin D biology, this review also discusses pharmacokinetic and pharmacodynamic considerations, potential biomarkers of treatment response, safety issues, and the current limitations of genotype- or epigenotype-guided vitamin D supplementation in male infertility.

## 2 Molecular distribution of vitamin D components within the male reproductive system

To evaluate local vitamin D signaling without overextending systemic associations, it is useful to first map VDR and key metabolic enzymes across the male reproductive tract. Human studies and focused reviews support the presence of vitamin D-related signaling components in the testis, epididymis, accessory glands, and mature spermatozoa (De Angelis et al., 2017; Gaml-Sørensen et al., 2023; Blomberg Jensen et al., 2010). This distribution provides an anatomical basis for local signaling, but the strength of functional evidence differs among tissues.

### 2.1 Testicular expression and local metabolism

Evidence for a testicular vitamin D axis comes from direct tissue studies, focused reviews, and broader human reproductive investigations (De Angelis et al., 2017; Gaml-Sørensen et al., 2023; Blomberg Jensen et al., 2010). Immunohistochemical and molecular analyses have localized VDR in developing germ cells, Sertoli cells, and Leydig cells, while expression of CYP27B1 and CYP24A1 supports the capacity for local activation and catabolism of vitamin D (Blomberg Jensen et al., 2010). This local machinery provides an anatomical basis for autocrine/paracrine vitamin D signaling during spermatogenesis and steroidogenesis, although cell-type-specific activity and physiological concentrations in the human testis remain incompletely defined.

### 2.2 Epididymis and accessory sex glands

The epididymis and accessory glands provide the luminal microenvironment for sperm maturation and transport. In human tissue, VDR together with vitamin D-metabolizing enzymes has been demonstrated in the epididymis, seminal vesicles, and prostate epithelium (Blomberg Jensen et al., 2010). These findings support a potential local role in epithelial and secretory function and in epididymal sperm maturation. However, direct functional studies in these accessory tissues remain limited, so proposed effects on seminal-fluid composition should be regarded as plausible rather than established.

### 2.3 Localization in mature spermatozoa

**FIGURE 1:**
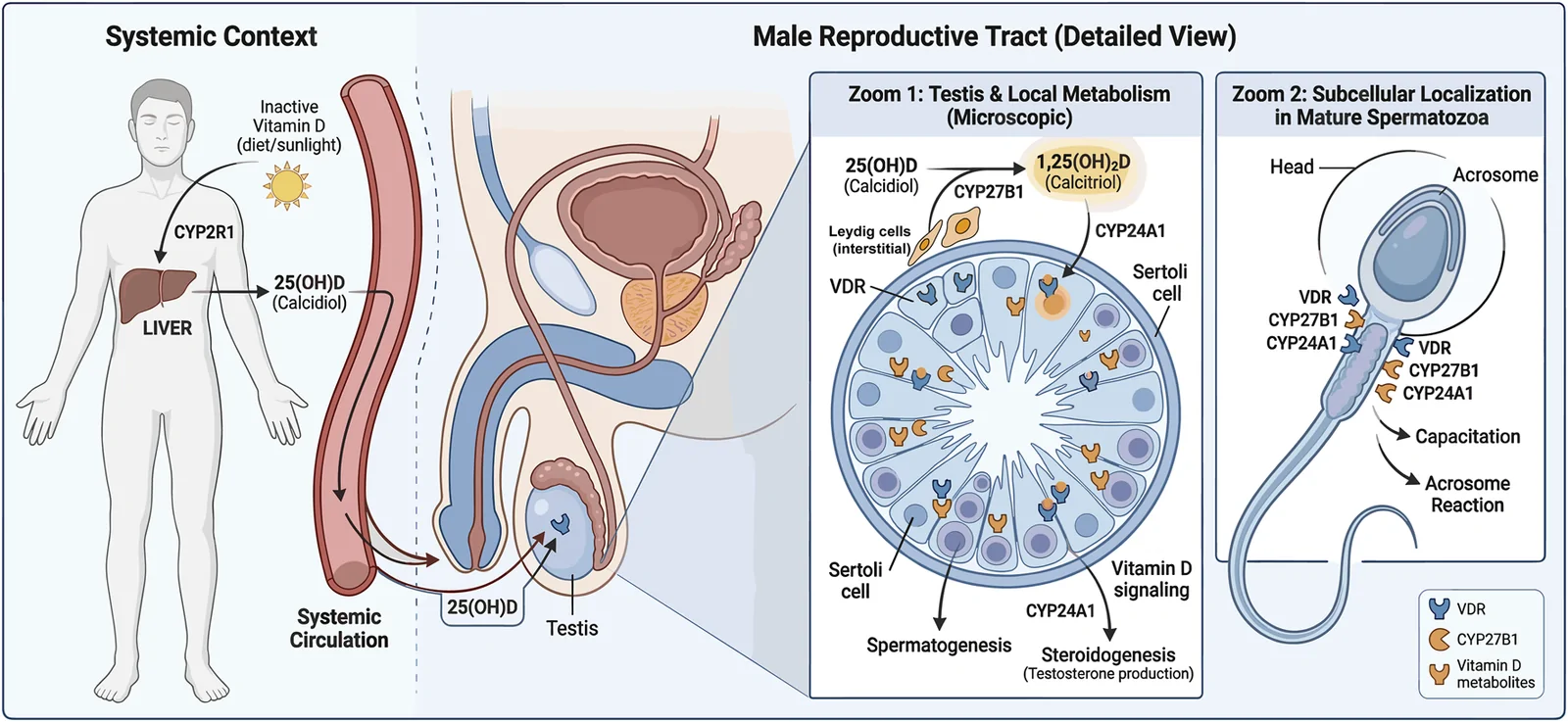
Systemic and localized metabolism and distribution of vitamin D components in the male reproductive tract. The figure illustrates the dual source of vitamin D signaling in male reproduction. Following systemic synthesis via hepatic CYP2R1, circulating 25(OH)D enters the male reproductive tract. Zoom 1 highlights the localized intrinsic metabolic network within the testis. Leydig cells are shown in the interstitial compartment outside the seminiferous tubules, whereas Sertoli cells and developing germ cells are located within the seminiferous epithelium. These cells express VDR and key vitamin D-metabolizing enzymes, including CYP27B1 and CYP24A1, enabling local synthesis and degradation of 1,25(OH)_2_D. Zoom 2 schematically depicts sperm-localized VDR, CYP27B1, and CYP24A1, emphasizing the head/acrosomal region; published studies have also reported neck/midpiece localization, indicating method- and subpopulation-dependent heterogeneity. This localization provides a structural basis for direct vitamin D-responsive actions on sperm function. Abbreviations: VDR, vitamin D receptor; CYP2R1, 25-hydroxylase; CYP27B1, 1α-hydroxylase; CYP24A1, 24-hydroxylase; 25(OH)D, 25-hydroxyvitamin D; 1,25(OH)_2_D, 1,25-dihydroxyvitamin D.

VDR and vitamin D-metabolizing enzymes have also been detected directly in mature human spermatozoa. Foundational human studies localized VDR predominantly to the sperm head/nuclear region with some neck labeling and demonstrated sperm expression of local vitamin D metabolic machinery (Blomberg Jensen et al., 2010; Aquila et al., 2008; Aquila et al., 2009; Calagna et al., 2022). Functional experiments further showed that 1,25(OH)_2_D_3_ can influence capacitation-related processes, intracellular Ca^2+^ signaling, motility, and acrosin activity through VDR-dependent mechanisms (Aquila et al., 2008; Aquila et al., 2009). Thus, the sperm-localized vitamin D system provides a plausible basis for direct regulation of fertilization-related events, while precise subcellular localization should not be considered uniform across studies. The specific cellular localization and proposed functions of these components are summarized in Table 1, and their systemic/local distribution is illustrated in Figure 1.

**TABLE 1:** Localization and pharmacological functions of vitamin D components in the male reproductive system.

Tissue/Organ	Specific cellular localization	Vitamin D components expressed	Proposed pharmacological/Biological functions
Testis	Leydig cells, Sertoli cells, and developing germ cells	VDR, CYP27B1 (1α-hydroxylase), CYP24A1 (24-hydroxylase)	Local synthesis and tight regulation of active 1,25(OH) _2_D_3_; maintains the microenvironment for spermatogenesis and steroidogenesis
Epididymis	Epididymal epithelial cells	VDR; vitamin D-metabolizing enzymes reported in human tissue	Potential regulatory role in the intricate process of sperm maturation during epididymal transit
Accessory Sex Glands	Prostate epithelial cells and seminal vesicles	VDR; vitamin D-metabolizing enzymes reported in human tissue	Modulation of the composition and secretory functions of the seminal plasma
Mature Spermatozoa	Reported in the head/acrosomal region and neck/midpiece, with study-dependent heterogeneity	VDR, CYP2R1, CYP27B1, CYP24A1	Direct and rapid modulation of specific sperm functions, including capacitation and the acrosome reaction

## 3 Molecular and pharmacological mechanisms modulating sperm function

Given the distribution of VDR and vitamin D-metabolizing enzymes across the male reproductive system, vitamin D signaling can be organized into two interacting modes: rapid non-genomic responses and slower VDR/RXR-dependent transcriptional regulation. Rather than treating these as separate mechanisms, the following sections link them to a limited set of reproductive endpoints, including calcium-dependent sperm activation, redox and mitochondrial homeostasis, steroidogenic support, and germ-cell survival (Dwivedi et al., 2025; Saini et al., 2025).

At the molecular level, vitamin D signaling is better conceptualized as an integrated receptor network than as a single linear pathway (Christ et al., 2016; Hanel et al., 2020; Blomberg, 2014). Circulating 25(OH)D can be converted to 1,25(OH)_2_D by CYP27B1 in classical endocrine tissues and within parts of the male reproductive tract (Bikle, 2014; Blomberg Jensen et al., 2010; Calagna et al., 2022). Ligand-activated VDR-RXR complexes regulate VDRE-containing genes involved in calcium handling, oxidative-stress control, immune modulation, steroidogenic support, and cell survival (Christ et al., 2016; Hanel et al., 2020; Meyer and Pike, 2013; Vázquez-Lorente et al., 2024; Basalamah et al., 2018). In parallel, human sperm studies support rapid non-genomic responses, including calcium mobilization and fertilization-related functional changes (Aquila et al., 2008; Aquila et al., 2009; Blomberg Jensen et al., 2011). These pathways converge on capacitation, mitochondrial/redox homeostasis, inflammatory control, steroidogenic support, and cell survival, although the direct evidence varies substantially across human, animal, and non-reproductive models (Blomberg, 2014; Blomberg Jensen et al., 2011; Alahmar, 2019; Yang et al., 2025; Xu et al., 2024; Hofer et al., 2014; Kim et al., 2005).

### 3.1 Calcium ion regulation and non-genomic actions

Calcium ions (Ca^2+^) are central to sperm hyperactivation, capacitation, and the acrosome reaction. Human sperm studies have identified VDR-related signaling and calcium-sensitive pathways and have shown that exposure to active 1,25(OH)_2_D_3_ can rapidly increase intracellular Ca^2+^, enhance motility, and promote acrosome-related responses (Aquila et al., 2008; Aquila et al., 2009; Blomberg Jensen et al., 2011). The speed of these responses supports a non-genomic, membrane-initiated component of vitamin D signaling in mature spermatozoa (Blomberg, 2014; Blomberg Jensen et al., 2011).

### 3.2 Antioxidant defense and mitochondrial function

Oxidative stress (OS) is a major pathological mechanism in male infertility, with elevated sperm oxidative stress reported in a substantial proportion of infertile men (Alahmar, 2019; Kaltsas, 2023). Vitamin D should not be characterized as a classical direct ROS-scavenging antioxidant. Mechanistically, vitamin D signaling may reduce oxidative burden indirectly by supporting endogenous antioxidant defenses, including SOD, GPx, and CAT, limiting NADPH oxidase-related pro-oxidant activity, and promoting antioxidant/inflammatory regulatory pathways such as Nrf2-related responses (Vázquez-Lorente et al., 2024; Tagliaferri et al., 2019). Its role in this context is therefore better framed as modulation of cellular redox homeostasis rather than direct chemical scavenging of ROS.

These mechanistic proposals are broadly consistent with clinical association studies reporting that lower systemic vitamin D can coexist with reduced antioxidant reserve and greater oxidative-stress markers in seminal plasma (Shahid et al., 2021). Comparative evaluations, for example, have noted that individuals dealing with infertility often present with depressed circulating vitamin D, paired with reduced total antioxidant capacity (TAC) and heightened concentrations of malondialdehyde (MDA) (Sepidarkish et al., 2019; Aşir et al., 2024). Multiple regression analyses further highlight vitamin D as an independent predictor for both MDA and TAC. Beyond its effects on redox balance, vitamin D signaling is tentatively linked to the optimization of ATP generation and the stabilization of mitochondrial membrane potential, both of which are critical for maintaining continuous sperm movement (Yang et al., 2025).

Inflammation also intersects with redox injury in the testis and spermatozoa. Activated Sertoli cells, testicular macrophages, and other immune cells can release pro-inflammatory cytokines such as TNF-α, IL-6, and IL-1β, which impair the blood-testis barrier and amplify ROS generation. Because spermatozoa have limited cytoplasmic antioxidant reserves and depend on mitochondrial ATP production for progressive motility, cytokine-driven ROS may damage the sperm plasma membrane, mitochondrial membrane potential, and nuclear DNA. Experimental testicular injury models support this link: vitamin D3 supplementation attenuated lead- or cisplatin-induced testicular damage, reduced TNF-α/IL-1β and NF-κB/iNOS signaling, restored antioxidant markers, and preserved seminiferous tubule architecture (Basalamah et al., 2018; Xu et al., 2024; Elrashidy et al., 2022). These findings support the hypothesis that vitamin D may protect sperm mitochondrial function partly through anti-inflammatory, NF-κB-related pathways, although direct confirmation in human sperm biology remains limited.

### 3.3 Hormonal and genomic regulation of spermatogenesis

Beyond rapid non-genomic actions, vitamin D operates as a classical nuclear receptor ligand. After ligand binding, VDR heterodimerizes with RXR and binds vitamin D response elements to modulate transcriptional programs relevant to steroidogenesis, germ-cell survival, and spermatogenic support. In human adult primary testicular cells, 1,25(OH)_2_D_3_ regulated 63 genes, including genes involved in androgen metabolism and vitamin D metabolism, and significantly increased testosterone synthesis *in vitro* (Hofer et al., 2014). In rodent testicular injury models, vitamin D_3_ restored the expression of steroidogenesis-related transcripts such as *Star*, *Cyp11a1*, *Hsd3b*, and *Hsd17b* (Elrashidy et al., 2022). Vitamin D3 has also been reported to modulate germ-cell proliferation and apoptosis markers, including increased PCNA/GCNA staining and BCL2 expression, reduced BAX and active caspase-3 expression, and fewer TUNEL-positive germ cells in aged testes (Jeremy et al., 2019). Consistently, vitamin D supplementation was recently shown to stimulate germ-cell proliferation in a restraint-stressed mouse model (Kumar et al., 2025). Therefore, current evidence links VDR signaling with steroidogenesis, apoptosis control, and spermatogenic maintenance, although direct ChIP-based evidence identifying these genes as VDR targets in human germ cells remains sparse.

### 3.4 Pharmacogenomics: the role of *VDR* gene polymorphisms

When evaluating biological responsiveness to vitamin D in the male reproductive system, an important source of inter-individual variability is genetic diversity in the *VDR* gene. Situated on chromosome 12q12-14, this highly polymorphic gene contains variants that may modify downstream transcriptional efficiency, receptor expression, ligand responsiveness, or receptor structure (Moradkhani et al., 2024). From a pharmacogenomic perspective, these polymorphisms may elucidate why a subset of infertile men exhibits an impaired reproductive response despite maintaining supposedly adequate systemic 25(OH)D levels.

Among the most extensively studied *VDR* single nucleotide polymorphisms (SNPs) are *FokI*, *BsmI*, *ApaI*, and *TaqI* (Chen et al., 2020). Researchers pay special attention to the FokI variation due to its position at the exon 2 translation initiation point. Specifically, while the “f” allele employs the primary start codon to generate a complete 427-amino-acid receptor, the “F” allele triggers translation further downstream, resulting in a slightly shortened protein of 424 amino acids. Current hypotheses cautiously suggest that this truncated “F” version might display superior interaction with the basal transcription apparatus, thereby exerting stronger transcriptional control over target genes than its full-length counterpart (Cao et al., 2016; Wang et al., 2021). Consequently, men carrying specific *FokI* genotypes might require different thresholds of circulating vitamin D to achieve the same molecular activation necessary for optimal sperm capacitation and motility.

Meanwhile, variations such as *TaqI*, *ApaI*, and *BsmI* are situated close to the 3′untranslated region (3′-UTR). Although they leave the receptor’s amino acid makeup unchanged, these specific mutations are believed to be intimately involved in governing both translation efficiency and the stability of *VDR* mRNA (Colombini et al., 2016; Castillo-Avila et al., 2021). Such regulatory shifts might ultimately reduce the total pool of VDR molecules capable of binding ligands within key sites like fully developed sperm and testicular tissue.

The integration of *VDR* gene polymorphisms into the molecular framework of male infertility offers one possible explanation for the heterogeneous outcomes observed in clinical supplementation trials. More broadly, genetic variation in vitamin D-related pathways may influence serum 25(OH)D concentrations, receptor activity, and tissue responsiveness to supplementation. A systematic review and meta-analysis reported that selected *VDR* variants, including TaqI and FokI, may modify the biochemical response to vitamin D supplementation (Usategui-Martín et al., 2022). However, the clinical utility of *VDR* genotyping in male infertility remains investigational. Current evidence does not yet define validated genotype-specific thresholds for serum 25(OH)D, sperm outcomes, or vitamin D dosage. Moreover, available studies are limited by ethnic heterogeneity, small sample size, linkage disequilibrium across loci, differences in baseline vitamin D status, and inconsistent reproductive endpoints. Therefore, *VDR*-guided vitamin D therapy should currently be regarded as a research direction rather than a routine clinical strategy for infertile men.

### 3.5 Epigenetic regulation and chromatin remodeling

Beyond direct genomic transcription and pharmacogenomic variations, the molecular effects of vitamin D on male fertility may also intersect with epigenetic networks. The production of sperm is an intricately coordinated event demanding substantial chromatin reorganization, which encompasses the crucial histone-to-protamine exchange, DNA methylation processes, and post-translational histone modifications (Cui et al., 2025). Rather than simply acting as a conventional transcriptional trigger, the VDR is increasingly recognized as a flexible structural regulator of the cell’s epigenome.

From a general nuclear receptor perspective, liganded VDR-RXR can recruit chromatin-regulatory machinery to vitamin D-responsive loci. Studies in non-testicular cell systems have shown that VDR signaling may involve p160/SRC-family coactivators, CBP/p300-containing histone acetyltransferase complexes, mediator/DRIP components, and, in some contexts, NCoR/SMRT-HDAC-containing corepressor complexes (Meyer and Pike, 2013; Kim et al., 2005). However, direct evidence that these exact VDR-associated coactivators or corepressors regulate spermatogenesis-specific genes in human testicular germ cells remains limited. Thus, in the male reproductive context, VDR-mediated chromatin remodeling should be interpreted as a biologically plausible mechanism extrapolated from broader VDR biology, rather than as a fully established germ-cell-specific pathway. This distinction is important because spermatogenesis involves unique chromatin remodeling events, including histone-to-protamine exchange, DNA methylation remodeling, and stage-specific histone modifications.

An additional layer of complexity is that the *VDR* gene itself may be subject to epigenetic regulation in male infertility. Clinical studies have reported altered methylation of the *VDR* promoter or seminal *VDR* gene methylation in men with idiopathic infertility, together with differences in serum vitamin D status and semen parameters (Hussein et al., 2021; Vladoiu et al., 2017). These findings suggest that epigenetic variation at the *VDR* locus may modify tissue responsiveness to vitamin D. However, causality remains uncertain, and it is not yet established whether normalization of serum 25(OH)D alone can reverse such epigenetic alterations or restore VDR-dependent signaling in reproductive tissues.

Vitamin D may also influence the sperm epigenetic landscape indirectly through redox and inflammatory pathways (Kowalówka et al., 2025; Carlberg, 2019). Oxidative stress is closely linked to sperm DNA damage, abnormal chromatin packaging, and altered DNA methylation patterns. Therefore, by modulating oxidative stress, mitochondrial function, inflammation, and steroidogenic signaling, vitamin D may help maintain a cellular environment favorable for normal sperm chromatin integrity. Nevertheless, evidence that vitamin D directly regulates specific DNA demethylases, histone acetyltransferases, or histone deacetylases in human spermatozoa or germ cells remains scarce.

**FIGURE 2:**
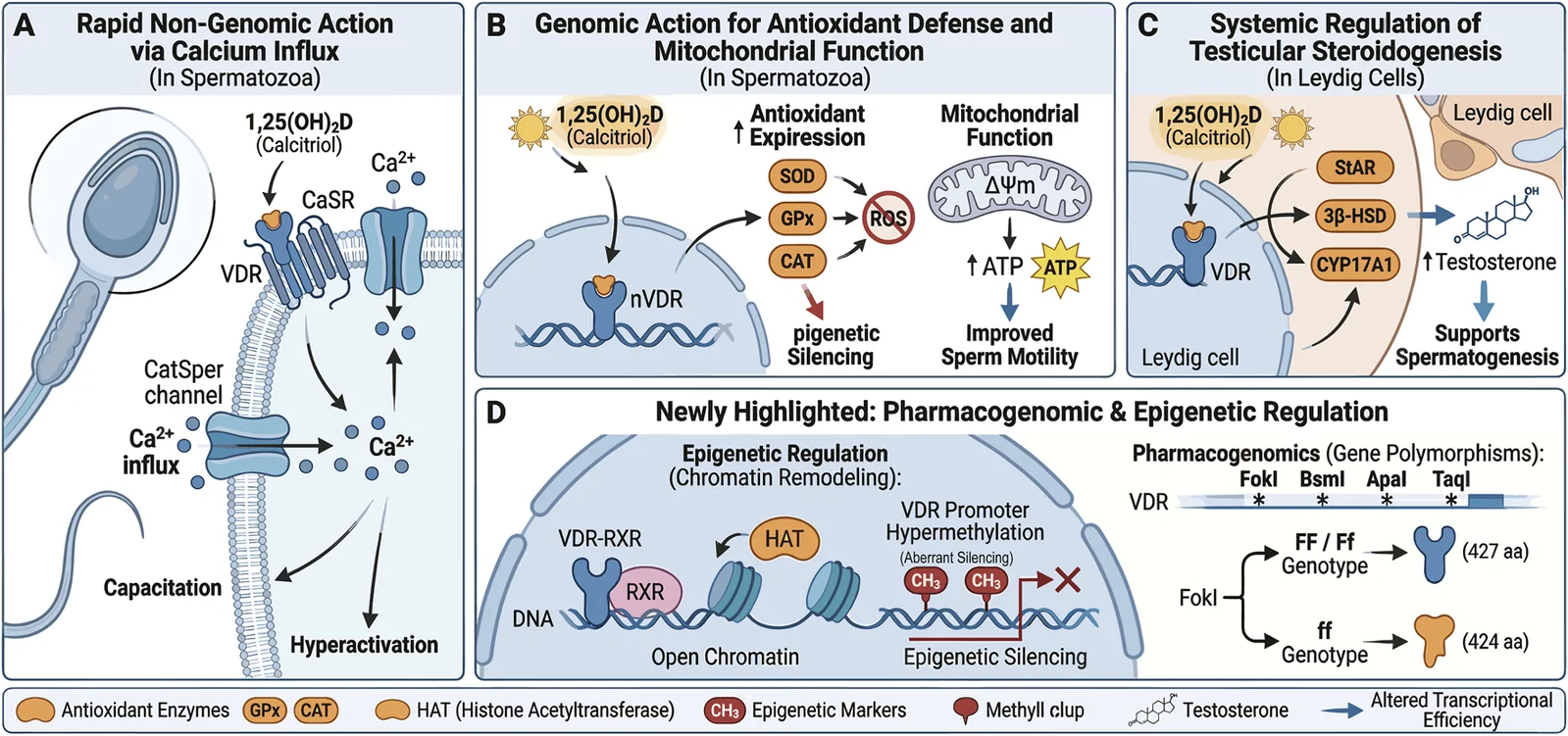
Comprehensive molecular, pharmacogenomic, and epigenetic mechanisms of vitamin D in male reproduction. This overarching mechanistic map delineates the multifaceted pathways through which the active metabolite, 1,25(OH)_2_D, exerts its effects. **(A)** Non-genomic actions: Rapid intracellular calcium (Ca^2+^) influx via membrane-associated VDR/CaSR complexes promotes sperm capacitation and hyperactivation. **(B)** Genomic antioxidant defense: Nuclear VDR signaling upregulates endogenous antioxidant enzymes (SOD, GPx, CAT) to mitigate reactive oxygen species (ROS) accumulation and preserve mitochondrial membrane potential (ΔΨm). **(C)** Steroidogenesis: Systemic and localized regulation of testosterone production in Leydig cells to support spermatogenesis. **(D)** Pharmacogenomic and epigenetic modulation: The activity and clinical relevance of these pathways may be modified by individual genetic and epigenetic landscapes. Right: *VDR* gene polymorphisms (e.g., *FokI*, *BsmI*, *ApaI*, *TaqI*) may alter receptor structure, expression, or transcriptional efficiency (e.g., *FokI* producing functionally distinct 427 aa vs. 424 aa VDR variants). Left: Epigenetic regulation is presented as a proposed mechanism. VDR-RXR complexes may recruit chromatin-regulatory cofactors in a cell-context-dependent manner, as shown mainly in non-testicular systems, whereas aberrant methylation of the *VDR* promoter may reduce receptor accessibility and modify tissue responsiveness to vitamin D. (Abbreviations: VDR, Vitamin D Receptor; CaSR, Calcium-Sensing Receptor; ROS, Reactive Oxygen Species; SOD, Superoxide Dismutase; GPx, Glutathione Peroxidase; CAT, Catalase; ATP, Adenosine Triphosphate; HAT, Histone Acetyltransferase; aa, amino acids.)

Taken together, current evidence supports a cautious model in which vitamin D signaling may interact with epigenetic regulation at two levels: first, through potential VDR-associated chromatin remodeling at vitamin D-responsive loci; and second, through epigenetic regulation of the *VDR* gene itself. This model may help explain inter-individual differences in response to vitamin D supplementation, but it remains an emerging hypothesis rather than an established therapeutic framework. Future studies using ChIP-seq, ATAC-seq, methylome profiling, and single-cell transcriptomic approaches are needed to identify *VDR*-associated regulatory loci in Sertoli cells, Leydig cells, germ cells, and mature spermatozoa. The integrated and partly proposed molecular, pharmacogenomic, and epigenetic mechanisms through which vitamin D may modulate male reproductive function are summarized in Figure 2.

Across model systems, the strongest direct human mechanistic evidence concerns VDR/vitamin D-metabolizing enzyme localization in the male reproductive tract and rapid Ca^2+^-dependent functional responses in mature sperm, with additional direct human testicular-cell evidence for steroidogenic effects. In contrast, antioxidant/anti-inflammatory protection, mitochondrial preservation, germ-cell survival/proliferation, and adverse effects of excessive vitamin D exposure are supported mainly by animal or other preclinical models, whereas reproductive epigenetic remodeling and genotype-guided treatment remain provisional. Table 2 summarizes these mechanisms according to the current strength of evidence.

**TABLE 2:** Strength of evidence for proposed vitamin D mechanisms in male reproduction.

Mechanism/pathway	Evidence category	Primary evidence base	Interpretation/key limitation
Local VDR and vitamin D-metabolizing machinery in the testis, reproductive tract, and mature spermatozoa	Well established	Direct human tissue and sperm localization studies (Blomberg Jensen et al., 2010; Aquila et al., 2008; Aquila et al., 2009; Calagna et al., 2022)	Provides a robust anatomical and biochemical basis for local vitamin D signaling; tissue-specific functional magnitude remains variable
Rapid Ca2+ signaling with effects on capacitation, motility, and acrosome-related responses	Well established	Direct functional studies in human spermatozoa (Aquila et al., 2008; Aquila et al., 2009; Blomberg Jensen et al., 2011)	Represents the strongest direct human functional mechanism, although most evidence is *ex vivo*/*in vitro*
VDR/RXR-dependent steroidogenic support	Supported by animal/preclinical studies	Human primary testicular-cell data plus rodent models (Hofer et al., 2014; Elrashidy et al., 2022)	Steroidogenic effects are supported experimentally, but *in vivo* human reproductive significance and germ-cell-specific VDR targets remain incompletely defined
Antioxidant/redox and anti-inflammatory protection	Supported by animal/preclinical studies	Rodent testicular-injury models, broader redox studies, and human association data (Vázquez-Lorente et al., 2024; Basalamah et al., 2018; Xu et al., 2024; Tagliaferri et al., 2019; Shahid et al., 2021; Sepidarkish et al., 2019; Aşir et al., 2024)	Biologically coherent, but direct causal confirmation in human spermatozoa or testicular tissue is limited
Mitochondrial preservation and ATP support relevant to sperm motility	Supported by animal/preclinical studies	Zebrafish and other mechanistic evidence (Yang et al., 2025)	Direct validation of vitamin D-dependent mitochondrial effects in human sperm remains limited
Germ-cell survival/proliferation and apoptosis control	Supported by animal/preclinical studies	Rat and mouse models (Jeremy et al., 2019; Kumar et al., 2025)	Direct evidence in human germ cells is sparse
Adverse reproductive effects of excessive vitamin D exposure/biphasic response	Supported by animal/preclinical studies	High-dose mouse model (Nasraldin et al., 2025)	Human reproductive toxicity has not been established; these findings should be distinguished from established systemic vitamin D toxicity (Office of dietary supplements N I O H, 2025)
VDR polymorphism-guided response and genotype-specific vitamin D thresholds/dosing	Hypothetical currently or requiring further validation	Human genetic association and supplementation-response meta-analyses (Moradkhani et al., 2024; Usategui-Martín et al., 2022)	No validated genotype-specific 25(OH)D thresholds, sperm-response criteria, or dosing strategies exist for male infertility
VDR-mediated reproductive epigenetic/chromatin remodeling	Hypothetical currently or requiring further validation	Non-testicular VDR chromatin studies plus human VDR methylation associations (Meyer and Pike, 2013; Kim et al., 2005; Hussein et al., 2021; Vladoiu et al., 2017; Kowalówka et al., 2025; Carlberg, 2019)	Direct germ-cell-specific chromatin occupancy and functional evidence remain limited

## 4 Clinical implications and translational pharmacology

Despite the compelling molecular framework suggesting that vitamin D is integral to male reproductive physiology, translating these *in vitro* pharmacological mechanisms into effective *in vivo* clinical therapies remains a significant challenge. The current clinical landscape is characterized by substantial observational data but conflicting interventional outcomes.

### 4.1 Correlation between serum vitamin D and semen parameters

Observational studies examining how routine semen metrics correspond to systemic vitamin D profiles—generally measured through circulating 25(OH)D—have produced somewhat mixed findings. However, the most reproducible link appears to involve the movement capabilities of sperm (Ciccone et al., 2021). An extensive analysis conducted by Cito and colleagues highlighted that a large proportion of their assessed research (seven out of ten cohorts) documented a favorable relationship connecting circulating vitamin D to forward sperm progression (Cito et al., 2020). This aligns mechanistically with the hypothesized non-genomic regulation of calcium influx required for flagellar hyperactivation (Blomberg Jensen et al., 2011).

Definitions of vitamin D deficiency, insufficiency, and severe deficiency are not uniform across studies or clinical guidelines. Accordingly, this review retains study-specific 25(OH)D cutoffs and states them when relevant rather than imposing a single universal threshold. For orientation, some infertility studies use <20 ng/mL (50 nmol/L) for deficiency, whereas the NIH/NASEM framework considers <12 ng/mL (<30 nmol/L) as indicating risk of deficiency and 12–20 ng/mL (30–50 nmol/L) as potentially inadequate; the 2024 Endocrine Society guideline does not endorse universal 25(OH)D thresholds for generally healthy adults (Office of dietary supplements N I O H, 2025; Demay et al., 2024).

On the other hand, attempting to correlate the hormone with total sperm output or overall concentration remains a highly debated topic (Arab et al., 2019). While some smaller cohorts report significantly higher sperm concentrations in vitamin D-sufficient men compared to deficient individuals (Derakhshan et al., 2020), comprehensive systematic reviews and meta-analyses often fail to replicate these findings. These findings suggest that vitamin D status may be more consistently related to sperm motility than to spermatogenic output, although even this relationship is influenced by baseline deficiency, study population, and analytical design.

### 4.2 Vitamin D status and sperm DNA fragmentation

Sperm DNA integrity is a critical determinant of male fertility, fertilization competence, embryo development, and pregnancy maintenance. The DNA fragmentation index (DFI) has therefore emerged as an important functional marker beyond conventional semen parameters. Because oxidative stress and mitochondrial dysfunction are major contributors to sperm DNA damage, vitamin D may theoretically influence DFI through antioxidant, anti-inflammatory, and mitochondrial-protective mechanisms.

Human evidence is currently based on only a small number of clinical studies and remains inconsistent. Blaseg et al. found no statistically significant difference in DFI across vitamin D-deficient, insufficient, and sufficient groups in a prospective cohort of men from infertile couples (Blaseg et al., 2022). By contrast, a retrospective cohort of men with varicocele reported an inverse association between serum vitamin D and DFI after adjustment for clinical and lifestyle factors, with a stronger association at lower vitamin D concentrations (Cai et al., 2025). These studies differ in underlying diagnosis, severity and distribution of vitamin D deficiency, DFI assay methodology, sample size, and adjustment for age, adiposity, lifestyle, abstinence interval, and other oxidative-stress-related factors. Such heterogeneity may contribute to the discrepant results and precludes a firm conclusion that serum 25(OH)D is an independent determinant of sperm DNA integrity (Blaseg et al., 2022; Cai et al., 2025).

Accordingly, vitamin D should not be presented as an established treatment for elevated DFI. In men with documented vitamin D deficiency, correction may be appropriate as part of general clinical care and comprehensive infertility management, but current evidence is insufficient to recommend supplementation specifically for the purpose of lowering DFI. Further prospective studies using standardized DFI assays and predefined vitamin D strata are needed.

### 4.3 Evaluating causality and the clinical efficacy of vitamin D supplementation

The critical clinical question is whether correcting vitamin D deficiency can meaningfully improve male factor infertility. To assess causality while reducing conventional observational confounding, Mendelian randomization (MR) studies have used genetic variants as instrumental variables. A large two-sample MR analysis reported an inverse association between genetically predicted 25(OH)D and male factor infertility risk (OR = 0.62) (Yuan et al., 2023).

Despite this genetic causal link, clinical interventional data from randomized controlled trials (RCTs) provide a more nuanced perspective on the therapeutic scope of supplementation. An exhaustive review conducted by Adamczewska et al. (2022) highlighted that although adding this secosteroid generally benefits the forward movement of sperm, most rigorously designed RCTs demonstrate that it fails to exert any significant modulatory effect on systemic reproductive hormones, including total testosterone (Adamczewska et al., 2022).

Looking past basic semen metrics to broader embryological success, a prominent secondary evaluation of a multicenter RCT (Banks et al., 2021) explored couples confronting mild male-driven infertility. Participants received either a placebo or a regimen containing 2,000 IU of vitamin D daily over a six-month period (Banks et al., 2021). Their findings indicated that while initial semen profiles, live birth frequencies, and overall pregnancy rates remained largely unaffected by the male’s vitamin D status, vitamin D deficiency (<20 ng/mL) correlated powerfully with an elevated incidence of miscarriage (adjusted OR 9.0) (Banks et al., 2021).

Taken together, these observations indicate that vitamin D is biologically relevant to male reproductive physiology, but the clinical benefit of supplementation remains context-dependent. MR evidence supports a potential long-term causal relationship between genetically influenced 25(OH)D status and male infertility risk, whereas RCTs evaluate short-term pharmacological correction and have not consistently demonstrated improvements in global semen parameters, reproductive hormones, pregnancy, or live birth. Therefore, vitamin D supplementation should not be interpreted as a universal infertility treatment. Its potential value may be greatest in selected deficient subgroups, particularly men with impaired motility, oxidative stress, elevated DFI, varicocele, obesity, or recurrent reproductive loss in the couple. The key study designs, pharmacological interventions, and clinical conclusions of these pivotal evaluations are summarized in Table 3. To provide historical context, Table 3 places selected foundational observational and functional studies alongside later randomized, genetic, and meta-analytic evidence.

**TABLE 3:** Selected landmark human evidence and clinical trial outcomes on vitamin D in male infertility.

Study	Study design	Population/exposure or intervention	Key clinical findings	Translational interpretation and limitations
Ramlau-Hansen et al. (2011)	Cross-sectional study	307 young healthy men; semen parameters and reproductive hormones were related to serum 25(OH)D	Low vitamin D was not clearly associated with poor semen quality; some crude adverse associations were observed at higher vitamin D levels and were sensitive to adjustment	An early human study showing that vitamin D-semen associations are not uniformly linear and may be confounded by population characteristics
Blomberg Jensen et al. (2011)	Cross-sectional study plus *in vitro* functional experiments	300 men from the general population for the clinical analysis; spermatozoa from additional donors were used for functional experiments	Serum vitamin D was positively associated with sperm motility; 1,25(OH)2D3 rapidly increased intracellular Ca2+, motility, and acrosome-related responses *in vitro*	A foundational study linking epidemiologic motility findings with a plausible rapid functional mechanism in human sperm
Hammoud et al. (2012)	Cross-sectional study	170 healthy men from the general population; semen and reproductive hormones were compared across serum 25(OH)D categories	Both low and high vitamin D categories showed lower selected semen measures compared with the intermediate range; reproductive hormones did not differ consistently	Provided early clinical support for a possible non-linear relationship rather than a simple deficiency-only model
Blomberg Jensen et al. (2018)	Triple-blinded randomized clinical trial	330 infertile men with 25(OH)D ≤ 50 nmol/L; vitamin D loading followed by 1,400 IU/day plus calcium for 150 days or placebo	Vitamin D/calcium did not improve semen quality overall; exploratory signals were observed for spontaneous pregnancy and live birth in selected subgroups	A landmark interventional trial demonstrating that correction of low vitamin D does not translate into broad semen improvement in an unselected infertile cohort
Banks et al. (2021)	Multicenter randomized controlled trial; secondary analysis	Men (n = 154) with mild male-factor infertility; participants received a formulation containing 2,000 IU/day vitamin D or placebo for up to 6 months	Baseline semen parameters, pregnancy rate, and live birth rate were not significantly improved; male vitamin D deficiency (<20 ng/mL) was associated with a higher risk of pregnancy loss	Suggests that vitamin D status may be more relevant to selected reproductive outcomes, such as pregnancy loss, than to routine semen parameters in unselected mild male-factor infertility
Blaseg et al. (2022)	Prospective cohort study	Men from infertile couples; serum vitamin D status and sperm DNA fragmentation index (DFI) were evaluated	No significant difference in DFI was observed among vitamin D-deficient, insufficient, and sufficient groups	Human evidence linking vitamin D to sperm DNA integrity remains inconsistent. Vitamin D status alone may not explain DFI variation in unselected infertile men
Adamczewska et al. (2022)	Systematic review	Review of clinical studies and randomized trials evaluating vitamin D and components of male fertility	Vitamin D supplementation showed possible benefits for sperm progressive motility, but no consistent effect on systemic reproductive hormones such as total testosterone	Supports a motility-centered rather than endocrine-centered interpretation of vitamin D supplementation. Evidence remains heterogeneous across populations and interventions
Yuan et al. (2023)	Two-sample Mendelian randomization study	Large-scale GWAS data evaluating genetically predicted serum 25(OH)D levels and male factor infertility risk	Higher genetically predicted 25(OH)D was associated with a reduced risk of male factor infertility (OR = 0.62)	Suggests a potential long-term causal relationship, but MR reflects lifelong genetically influenced vitamin D status rather than short-term supplementation
Tania et al. (2023)	Systematic review and meta-analysis of randomized clinical trials	Infertile men receiving vitamin D supplementation or placebo in randomized trials	Vitamin D supplementation was associated with improvements in total sperm motility, progressive motility, and normal morphology	Provides supportive interventional evidence, but the number of trials was limited and heterogeneity in dose, duration, baseline deficiency, and study population should be considered
Cai et al. (2025)	Retrospective cohort study	Men with varicocele; serum vitamin D and DFI were analyzed with adjustment for clinical and lifestyle factors	Serum vitamin D was inversely associated with DFI, with a stronger association at lower vitamin D concentrations	Vitamin D may be more relevant to sperm DNA integrity in oxidative-stress-prone subgroups, such as men with varicocele or documented deficiency. Causality cannot be inferred from retrospective data
Michaelsen et al. (2025)	Systematic review and meta-analysis of dietary supplements	Broad evaluation of dietary supplements for male infertility, including pregnancy, live birth, and sperm parameters	No convincing evidence showed that vitamin D improved sperm parameters; certainty of evidence for many supplement-related outcomes was low or very low	Highlights current uncertainty and supports a cautious clinical interpretation. Vitamin D should not be considered a universal treatment for male infertility

### 4.4 Why clinical evidence is inconsistent: baseline status, dose, genotype, and study design

The apparent discrepancies among observational studies, Mendelian randomization analyses, and randomized controlled trials likely reflect differences in study design and biological context rather than a simple contradiction. Observational studies capture real-world variation in sunlight exposure, diet, body mass index, ethnicity, seasonal changes, comorbidities, and lifestyle factors, but they are vulnerable to residual confounding and reverse causation. Mendelian randomization studies estimate the effect of lifelong genetically influenced differences in circulating 25(OH)D and may therefore reflect long-term biological exposure rather than the short-term effect of supplementation. In contrast, RCTs test a defined intervention within a limited time window and may fail to show benefit if participants do not have clearly low baseline 25(OH)D concentrations or if the intervention does not cover a complete spermatogenic cycle (Arab et al., 2019; Misell et al., 2006).

The two principal randomized datasets discussed here were also modest in size and clinically heterogeneous: Blomberg Jensen et al. enrolled 330 infertile men with 25(OH)D ≤ 50 nmol/L and used a loading regimen followed by 1,400 IU/day plus calcium for 150 days, whereas the Banks et al. secondary analysis included 154 men with mild male-factor infertility receiving a formulation containing 2,000 IU/day of vitamin D or placebo for up to 6 months (Banks et al., 2021; Blomberg Jensen et al., 2018). Differences in entry criteria, co-intervention, treatment duration, baseline vitamin D status, and outcome hierarchy limit direct comparison across these trials.

Several clinical factors may further explain the heterogeneous findings. First, baseline vitamin D status is critical: men with lower baseline 25(OH)D concentrations may be more likely to benefit from supplementation than those with sufficient or near-sufficient 25(OH)D concentrations. Second, dose and duration vary widely among trials, ranging from low daily doses to high weekly or loading regimens, often with different co-interventions such as calcium. Third, semen parameters are biologically variable and may require at least one full spermatogenic cycle to show measurable change. Fourth, ancestry, skin pigmentation, latitude, adiposity, dietary habits, and vitamin D-binding protein status may influence circulating and bioavailable 25(OH)D (Bikle and Schwartz, 2019; Wang et al., 2010). Fifth, genetic and epigenetic variability in the vitamin D pathway, including *VDR*, *CYP2R1*, *CYP27B1*, *CYP24A1*, and *GC*, may alter tissue responsiveness to the same serum 25(OH)D concentration (Calagna et al., 2022; Moradkhani et al., 2024; Hussein et al., 2021; Vladoiu et al., 2017; Kowalówka et al., 2025; Carlberg, 2019). Finally, many trials are underpowered for clinically meaningful endpoints such as pregnancy, miscarriage, live birth, and sperm DNA integrity.

Recent meta-analyses also illustrate this uncertainty. One meta-analysis of randomized clinical trials reported that vitamin D supplementation may improve total sperm motility, progressive motility, and normal morphology in infertile men (Tania et al., 2023). By contrast, a broader systematic review and meta-analysis of dietary supplements found no convincing evidence that vitamin D improved sperm parameters, and emphasized that the certainty of evidence for many supplements was low or very low (Michaelsen et al., 2025). These differences reinforce a precision-medicine interpretation: vitamin D supplementation is unlikely to benefit all infertile men equally, but it may be relevant in selected subgroups characterized by documented vitamin D deficiency, oxidative stress, asthenozoospermia, elevated DFI, varicocele, obesity, or specific vitamin D pathway variants.

### 4.5 Therapeutic window and safety considerations for excessive vitamin D exposure

Although vitamin D deficiency may be detrimental to male reproductive function, the relationship between vitamin D exposure and sperm quality is unlikely to be linear. The reproductive evidence for excess is currently predominantly preclinical. In adult male mice, high-dose vitamin D administration reduced sperm count and motility, lowered testosterone, decreased testicular size and weight, altered testicular histoarchitecture, and increased *CDKN1B* expression in spermatocytes, consistent with impaired cell-cycle progression during spermatogenesis (Nasraldin et al., 2025).

Human vitamin D toxicity is rare and is usually caused by excessive supplemental intake rather than sun exposure. Established toxicity is mediated mainly by hypercalcemia and hypercalciuria and can present with nausea, vomiting, muscle weakness, neuropsychiatric symptoms, dehydration, polyuria, nephrolithiasis, and, in severe cases, renal failure or soft-tissue calcification. For adults in the general population, 4,000 IU/day is the tolerable upper intake level for chronic intake; this is a population safety limit rather than a toxicity threshold. Frank toxicity is typically associated with much higher exposure and serum 25(OH)D concentrations above approximately 150 ng/mL (375 nmol/L) (Office of dietary supplements N I O H, 2025). Thus, in this review, “excessive exposure” should not be interpreted as synonymous with any intake above 4,000 IU/day or with clinically supervised pharmacological dosing.

These established human toxicities should be distinguished from the reproductive effects described in animal models. In the mouse study above, the adverse spermatogenic phenotype may involve disturbed calcium signaling, systemic toxicity and oxidative stress, steroidogenic suppression, and *CDKN1B*-related cell-cycle effects (Nasraldin et al., 2025; Office of dietary supplements N I O H, 2025). Direct human evidence that vitamin D excess impairs spermatogenesis is currently limited. Accordingly, supplementation should aim to restore physiological vitamin D homeostasis rather than maximize serum 25(OH)D concentrations; high-dose or prolonged regimens should be medically indicated and accompanied by appropriate monitoring of serum 25(OH)D, calcium, and renal function (Office of dietary supplements N I O H, 2025; Demay et al., 2024).

### 4.6 Translational pharmacology and pragmatic clinical recommendations

From a translational pharmacology perspective, vitamin D supplementation in male infertility should be guided by pharmacokinetic, pharmacodynamic, safety, and patient-stratification considerations. After oral intake or cutaneous synthesis, vitamin D is transported in circulation mainly bound to vitamin D-binding protein and albumin, hydroxylated in the liver to 25(OH)D, and subsequently converted to the active metabolite 1,25(OH)_2_D by CYP27B1 in the kidney and selected extra-renal tissues. Because circulating 25(OH)D has a longer half-life than 1,25(OH)_2_D and reflects body vitamin D stores, it remains the most practical clinical biomarker for assessing vitamin D status (Holick, 2007; Jones, 2008; Bikle, 2014; Office of dietary supplements N I O H, 2025). However, serum 25(OH)D does not necessarily capture local testicular metabolism, bioavailable vitamin D, or tissue-level VDR responsiveness.

The pharmacodynamic response to vitamin D is unlikely to be linear. A clinically relevant goal is restoration of vitamin D homeostasis rather than supraphysiological exposure. Potential response biomarkers in infertile men include serum 25(OH)D, calcium, parathyroid hormone, renal function, sperm progressive motility, seminal oxidative stress markers such as ROS, MDA, and TAC, and sperm DNA fragmentation index. In research settings, *VDR* polymorphisms, vitamin D pathway gene variants, and *VDR* promoter methylation may help identify subgroups with altered responsiveness, but these markers are not yet validated for routine clinical decision-making (Hussein et al., 2021; Vladoiu et al., 2017). In line with recent precision-medicine and nutrigenomic perspectives, vitamin D supplementation in male infertility should therefore be viewed as targeted correction of a biologically relevant deficiency within a broader VDR-centered response network, rather than as empirical supplementation for all infertile men.

**FIGURE 3:**
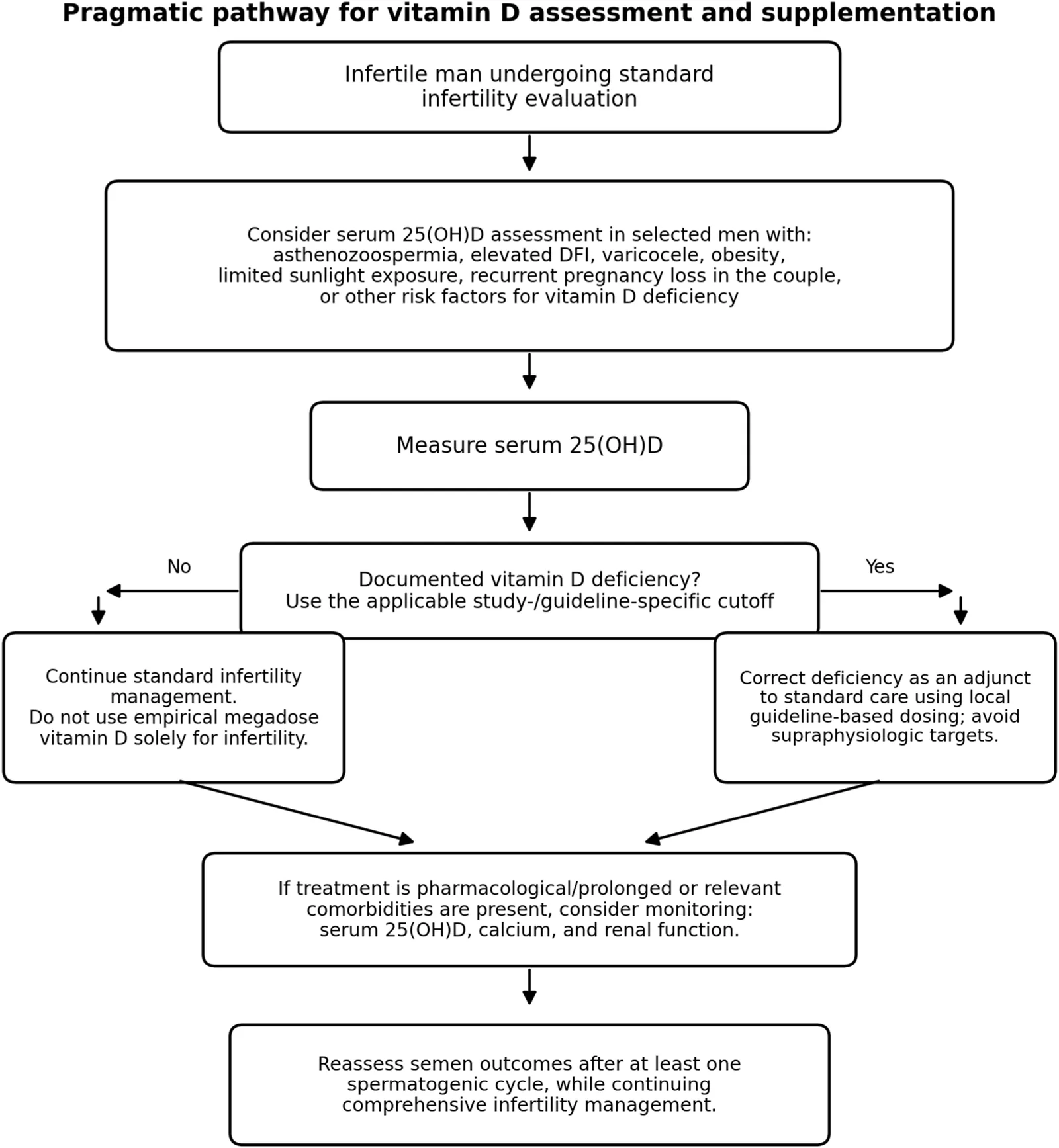
Pragmatic clinical pathway for considering vitamin D assessment and supplementation in infertile men. Serum 25(OH)D testing may be considered when infertility coexists with asthenozoospermia, elevated DFI, varicocele, obesity, limited sunlight exposure, recurrent pregnancy loss in the couple, or other recognized risk factors for vitamin D deficiency. If deficiency is documented using the applicable study- or guideline-specific threshold, vitamin D may be corrected as an adjunct to standard infertility management using locally recommended dosing, while empirical megadose supplementation and supraphysiologic targets should be avoided. For pharmacological or prolonged treatment, or in the presence of relevant comorbidities, monitoring of serum 25(OH)D, calcium, and renal function should be considered. Semen outcomes may be reassessed after at least one spermatogenic cycle. DFI, DNA fragmentation index; 25(OH)D, 25-hydroxyvitamin D.

In clinical practice, serum 25(OH)D assessment may be considered in infertile men with asthenozoospermia, elevated DFI, varicocele, obesity, limited sunlight exposure, recurrent pregnancy loss in the couple, or other risk factors for deficiency. When deficiency is confirmed, targeted supplementation may be used as an adjunct to comprehensive infertility management. Commonly used regimens generally fall within approximately 1,000–4,000 IU/day, depending on baseline vitamin D status, comorbidities, local guidelines, and safety monitoring (Office of dietary supplements N I O H, 2025; Demay et al., 2024). Treatment should ideally last long enough to cover at least one spermatogenic cycle before semen outcomes are reassessed. Empirical megadose supplementation should be avoided, especially in men without documented deficiency (Misell et al., 2006). A pragmatic pathway for when to consider 25(OH)D assessment and targeted supplementation is summarized in Figure 3.

Safety monitoring is important when pharmacological doses are used or when supplementation is prolonged. Serum 25(OH)D, calcium, and renal function should be considered in men receiving high-dose therapy, those using calcium co-supplementation, and those with renal disease, granulomatous disease, hyperparathyroidism, nephrolithiasis, or medications that interact with vitamin D metabolism. Overall, current evidence supports vitamin D correction as a low-cost and biologically plausible adjunct in deficient men, but not as a stand-alone treatment for male infertility or as an empirical intervention for all infertile men.

## 5 Limitations of current evidence and future research priorities

Several limitations should be acknowledged when interpreting the current literature. First, many mechanistic findings are derived from animal models or non-reproductive cell systems, and direct validation in human Sertoli cells, Leydig cells, germ cells, and mature spermatozoa remains limited. Second, human observational studies are susceptible to confounding by sunlight exposure, season, diet, adiposity, comorbidities, and lifestyle factors. Third, interventional studies vary substantially in baseline vitamin D status, dosage, treatment duration, calcium co-administration, ethnicity, infertility diagnosis, and semen endpoints. Fourth, most clinical trials are underpowered for pregnancy, miscarriage, live birth, and sperm DNA integrity outcomes. Fifth, the therapeutic window for male reproductive endpoints remains unclear, particularly given emerging evidence that excessive vitamin D exposure may also impair sperm parameters in animal models.

Future studies should prioritize well-powered, placebo-controlled trials that enroll clearly defined vitamin D-deficient infertile men and stratify participants by baseline 25(OH)D, obesity, oxidative stress status, varicocele, DFI, and vitamin D pathway genotypes. Trial duration should cover at least one spermatogenic cycle, and outcome measures should extend beyond routine semen parameters to include oxidative stress markers, mitochondrial function, DFI, pregnancy loss, clinical pregnancy, and live birth. Mechanistic studies should combine single-cell transcriptomics, methylome profiling, ChIP-seq, ATAC-seq, proteomics, and metabolomics to define the reproductive cell-specific VDR regulatory network. Such approaches may clarify whether vitamin D acts primarily as a motility-preserving homeostatic regulator, an antioxidant and anti-inflammatory adjunct, or a context-dependent pharmacological target in selected subgroups of infertile men.

## 6 Conclusion

Current evidence supports a biologically plausible role for vitamin D signaling in male reproduction. The presence of VDR and vitamin D-metabolizing enzymes in the male reproductive tract and mature spermatozoa provides an anatomical basis for local and systemic actions. Mechanistically, vitamin D may influence sperm function through calcium-dependent non-genomic signaling, antioxidant defense, mitochondrial regulation, inflammatory modulation, steroidogenic support, and transcriptional regulation. However, several proposed pathways, particularly those involving epigenetic remodeling and germ-cell-specific VDR target genes, still require direct validation in human reproductive tissues.

Clinically, low serum 25(OH)D has been most consistently associated with impaired sperm motility, whereas associations with sperm concentration, total sperm count, reproductive hormones, DFI, pregnancy, and live birth remain inconsistent. Mendelian randomization studies suggest a potential protective relationship between higher genetically predicted 25(OH)D and male infertility risk, but randomized trials have not established vitamin D supplementation as a broadly effective treatment for unselected infertile men. Moreover, emerging preclinical evidence indicates that excessive vitamin D exposure may also impair spermatogenesis, supporting a biphasic model of vitamin D homeostasis.

Therefore, vitamin D should be considered a homeostatic and context-dependent modulator rather than a stand-alone pharmacological treatment for male infertility. Assessment and correction of deficiency may be reasonable in selected men, particularly those with asthenozoospermia, oxidative stress, elevated DFI, varicocele, obesity, limited sun exposure, or recurrent reproductive loss in the couple. Future translational studies should define optimal therapeutic windows, identify validated biomarkers of response, and determine whether VDR-mediated molecular signaling, nutrient–gene interactions, vitamin D pathway genotypes, or epigenetic markers can guide individualized supplementation strategies.
